# Improving the immunosuppressive potential of articular chondroprogenitors in a three-dimensional culture setting

**DOI:** 10.1038/s41598-020-73188-9

**Published:** 2020-10-06

**Authors:** Guillermo Bauza, Anna Pasto, Patrick Mcculloch, David Lintner, Ava Brozovich, Federica Banche Niclot, Ilyas Khan, Lewis W. Francis, Ennio Tasciotti, Francesca Taraballi

**Affiliations:** 1 Center for NanoHealth, Swansea University Medical School, Swansea University Bay, Singleton Park, Wales, SA2 8PP UK; 2 Center for Musculoskeletal Regeneration, Houston Methodist Research Institute, 6670 Bertner Ave, Houston, TX 77030 USA; 3 Orthopedics and Sports Medicine, Houston Methodist Hospital, 6565 Fannin Street, Houston, TX 77030 USA; 4 Texas A&M College of Medicine, 8447 Highway 47, Bryan, TX 77807 USA; 5 Department of Applied Science and Technology, Polytechnic of Turin, Corso Duca degli Abruzzi 24, 10129 Turin, Italy

**Keywords:** Osteoimmunology, Mesenchymal stem cells

## Abstract

Cartilage repair in osteoarthritic patients remains a challenge. Identifying resident or donor stem/progenitor cell populations is crucial for augmenting the low intrinsic repair potential of hyaline cartilage. Furthermore, mediating the interaction between these cells and the local immunogenic environment is thought to be critical for long term repair and regeneration. In this study we propose articular cartilage progenitor/stem cells (CPSC) as a valid alternative to bone marrow-derived mesenchymal stem cells (BMMSC) for cartilage repair strategies after trauma. Similar to BMMSC, CPSC isolated from osteoarthritic patients express stem cell markers and have chondrogenic, osteogenic, and adipogenic differentiation ability. In an in vitro 2D setting, CPSC show higher expression of *SPP1* and *LEP*, markers of osteogenic and adipogenic differentiation, respectively. CPSC also display a higher commitment toward chondrogenesis as demonstrated by a higher expression of *ACAN*. BMMSC and CPSC were cultured in vitro using a previously established collagen-chondroitin sulfate 3D scaffold. The scaffold mimics the cartilage niche, allowing both cell populations to maintain their stem cell features and improve their immunosuppressive potential, demonstrated by the inhibition of activated PBMC proliferation in a co-culture setting. As a result, this study suggests articular cartilage derived-CPSC can be used as a novel tool for cellular and acellular regenerative medicine approaches for osteoarthritis (OA). In addition, the benefit of utilizing a biomimetic acellular scaffold as an advanced 3D culture system to more accurately mimic the physiological environment is demonstrated.

## Introduction

With a projected increase of 50% in the next 20 years, Osteoarthritis (OA) is one of the highest contributors to the global burden of disease^1^. OA is considered both a degenerative and inflammatory condition^2^, with local inflammation triggering or activating the post-traumatic healing cascade^3^. Endothelial cells, fibroblasts and especially immune cells such as natural killer (NK), cytotoxic T cells, macrophages, monocytes and Th1 CD4^+^ cells, release pro-inflammatory cytokines such as interferon gamma (IFN-y) and Tumor-necrosis factor alpha (TNF-a) that play a pivotal role in the pathogenesis of OA. These cytokines act as chemoattractants, attracting leukocytes, especially neutrophils, to the site of injury. Immune cell infiltration within the joint space results in the production and release of other proinflammatory mediators such as prostaglandin 2 (PGE2)^4^, as well as matrix-degrading enzymes and metalloproteinase (MMPs) that potentiate tissue damage^5,6^.

The cellular and molecular factors associated with initial inflammation combine to impair the physiological repair processes that are usually sustained by resident progenitor and/or mature resident chondrocyte cells, leading to chronic disease^7,11^. Addressing the trauma-induced imbalance between inflammatory and regenerative cells has been proposed as a critical step towards preventing or stopping the degenerative process that leads to OA progression^8^. As a result, a better understanding of the interplay between key components of the tissue microenvironment is needed to identify tailored regenerative approaches for this tissue niche, to prevent the development of this debilitating disease^9,10^. Though various cellular and acellular repair strategies have been proposed to date, the restoration of articular cartilage remains a challenge, in part, due to in vitro studies failing to accurately mimick the 3D tissue environment^11^.

In all acellular technologies, regeneration relies on the ability of resident stem and progenitor cells to repopulate the damaged area, integrate with the implanted material, and restore tissue homeostasis^12^. To date, bone marrow-derived mesenchymal stem cells (BMMSC), have been one of the main sources of mesenchymal stem cells (MSC) in the treatments of OA^13,14^. BMMSC not only have the ability to self-regenerate and differentiate, but also have enormous immunosuppressive potential^15,16^. BMMSCs act on macrophages, neutrophils, natural killer, dendritic cells, and adaptive immune cells (T and B cells) to modulate their anti/pro-inflammatory activity. They have also been shown to use paracrine signaling to induce the production of bioactive molecules that suppresses immune cell proliferation and maturation, induce inflammatory cell apoptosis, helping distinct tissue restoration processes^17^. However, these properties are transient and vary greatly depending on the specific tissue environment, representing one of the major limitations in the application of BMMSC for MSC-based therapies^18^.

In addition to BMMSC, cartilage progenitor/stem cells (CPSC) have been shown to reside at the tissue surface of the knee joint and migrate to the full depth of the tissue during development. CPSCs exhibit greater chondrogenic capacity compared to fully differentiated chondrocytes^19,20^, and in conditions such as OA, respond to inflammatory signals by migrating to the site of injury^21^. Their utilization and potential role in cartilage regeneration however, has not been fully investigated ^22^.

Thus, we decided to evaluate the immunomodulatory potential of human CPSC, compared to BMMSC, in an in vitro TNFa- and IFNy-mediated inflammation model. To overcome the lack of in vitro based immune modulatory assays that adequately replicate the human cartilage immunogenic environment in vivo*,* we exploited a chondroitin sulfate (CS) porous collagen scaffold (developed by our group) which resembles native cartilage tissue. The CS recapitulates the biological, structural, and functional features of the chondrogenic niche environment^22^. In addition, the scaffold also exhibits an immunomodulatory effect on native immune cells that infiltrate the scaffold, improving the overall regenerative outcome^37^. In vitro, the CS scaffold provides the basis for the 3D physiological niche mimicking culture conditions, enabling the maintenance of the stem/progenitor phenotype in long term culture^23,24^. Furthermore, it provides ideal, advanced 3D culture systems in which to interrogate BMMSC and CPSC cell populations to recapitulate the chondrogenic niche, direct differentiation, reorganize the ECM, and modulate inflammation^12^.

In this study, we suggest CPSC are anti-inflammatory immune modulators and enhancers of tissue repair^25^. The ability of human cartilage derived-CPSC to mediate the acute immune response was demonstrated in vitro using a biomimetic 3D collagen-CS scaffold model that mimics the chondrogenic niche. This study clearly demonstrates the utility of advanced 3D culture techniques in understanding the repair capacity of resident cartilage specific progenitor/stem cells in vitro, providing supportive evidence for the potential suitability of resident CPSC to treat focal chondral defects found in OA.

## Materials and methods

### Authors’ statement

All methods described were carried out in accordance with protocols approved by the Houston Methodist Institutional Review Board (IRB) to ensure the rights and welfare of human subjects protection during their participation compliance with the Code of Federal Regulations (45 CFR 46) established by Houston Methodist Research Institute with identification numbers CR00006624 and Pro00012800. Informed consent was obtained from all subjects participating in the study.

### Sample acquisition, cell isolation and cell culture

Human bone marrow aspirate (HBMA) or partial articular cartilage (AC) joints where obtained from the orthopedic biorepository at Houston Methodist Hospital Orthopedics and Sports Medicine Department (IRB CR00006624). A total of 31 patients, 19 males and 12 females with 50.19 ± 18.93 and 52.95 ± 16.06 years of age respectively were included after signed consent. The samples were individual and anonymously bio-banked with specific identification numbers. A minimum of three biological patient samples were used for each experiment (n = 3) unless otherwise specified in the experimental methods, with patient sample demographics age and sex matched in each case. 10 ml of HBMA were washed with a solution of PBS and 10 ml standard medium (α-MEM) supplemented with 15% fetal bovine serum (FBS) and 1% penicillin (100 UI/ml)-streptomycin (100 mg/ml), before seeding at 0.26 ml per cm^2^. AC of healthy appearance, avoiding spongy and fibrotic layers, was aseptically collected and enzymatically digested by incubating at 37 °C for 90 min with pronase (Sigma) and following by being treated with collagenase for 16 h (Sigma), both at 1 mg/ml, to yield all cellular material. Cells were then subjected to a differential adhesion fibronectin assay to specifically isolate CPSC, which were then seeded in standard media^51^. BMMSC have been obtained from HBMA as previously reported^26^. Both cellular types were expanded and cryobanked at 10^6^ cells per vial in 90% FBS plus 10% dimethyl sulfoxide (DMSO). Restored cells were seeded at 5 × 10^3^ cells per cm^2^ and cultured at least 24 h in standard media, before performing the experiments, at 37 °C in a humidified atmosphere (90%) with 5% CO_2_. For the 3D scaffold cultures, 20 μl of medium containing 2.5 × 10^5^ cells were seeded on the center of each collagenous scaffold (CSCL) and kept in an incubator for 30 min. Culture medium was then added to each well and medium was changed twice per week thereafter or according to the experiment design. Unless otherwise stated in specific experiments, all cells were expanded up to a maximum of 5 passages. In addition, 2D and 3D experiments were normally seeded simultaneously, so direct comparisons could be made.

### Cell doubling, colony formation efficiency and proliferation

Cell doubling time was evaluated over 10 in vitro culture passages seeding 10^4^ of BMMSC and CPSC into 24-well tissue culture polystyrene dishes as previously described^27^. Cells were trypsinized at 90% confluence, counted and plated again at the same density. The mean of population doublings was calculated at each passage according to the formula CD = log(Nc/No)/log2 and PD = CT/CD, where CD represents cell doubling, Nc represents the number of cells at confluence, No represents seeded cells and CT represents the culture time. To assess colony forming unit (CFU) efficiency, 50, 100, 200 and 500 BMMSC or CPSC were seeded in p100 polystyrene culture dishes and allowed to grow for 14 days. Fixation with 10% neutral buffered formalin (NBF) was followed by crystal violet (0.5 mg/ml) staining and visual counting of the colonies formed. Cell proliferation and cytotoxicity were evaluated by AlamarBlue assay (Invitrogen, ThermoFisher Scientific) according to manufacturer’s instructions over 72 h of culture. Optical density was measured at wavelengths of 570 and 600 nm. Because the culture medium was not changed during this period, the calculated percentage of AlamarBlue reduction (%AB) is a cumulative value. Values are reported as %AB over time, which is associated with the presence of metabolically active cells. Data representative of three independent experiments were reported.

### Multilineage differentiation

Multilineage cell potential towards osteogenic, adipogenic, and chondrogenic differentiation was assessed in vitro at P4. BMMSC and CPSC were seeded at the density of 5,000 cells/cm^2^ in 12-well plates and cultured until approximately 80–90% of confluence. Then the media was replaced according to the differentiation assay using StemPro Chondrogenesis, Osteogenesis or Adipogenesis Differentiation Kit (all from Gibco) for Chondrogenic, Osteogenic or Adipogenic induction, respectively. At different time points (7, 14 and 21 days) the multilineage potential was evaluated using different staining protocols. For chondrogenic differentiation conventional Alcian blue staining (Sigma) was performed to highlight proteoglycans deposition. Osteogenic induction was confirmed evaluating mineral deposition with von Kossa staining (Von Kossa Stain Kit American MasterTech). ImageJ software was used to quantify Von Kossa stained sample images. For adipogenic induction, intracellular lipid droplets accumulation was visualized by Oil red O staining (Oil Red O Stain Kit American MasterTech). For both BMMSC and CPSC, non-induced cells were used as control and cultured for the same time in normal medium^27^.

### Flow cytometry analysis

BMMSC and CPSC were collected and characterized for the expression of MSC-associated markers. Briefly, cells from 2D cultures were recovered using trypsin whereas cells seeded into 3D scaffolds were first subjected to partial digestion (30 min) with collagenase 2 mg/ml at 37 °C. The cells were then washed with FACS buffer and stained for 30 min at 4 °C with the following antibodies: FITC anti-mouse/human CD90, APC anti-human CD73, and PerCP anti-human CD105 (all from BioLegend) and negative anti-human HSC cocktail (PE CD45, PE CD34, PE CD11b, PE CD19 and PE HLA-DR, BD analysis kit). Cells were detected using BD LSR Fortessa (Beckton Dickinson) and data were analyzed with FLowJo (10.0.7) software.

### Immunosuppressive potential stimulation

For in vitro cell stimulation, BMMSC and CPSC were seeded at the density of 5 × 10^3^ cells/cm^2^ in 24-well plates in standard supplemented medium, α-MEM supplemented with 15% fetal bovine serum (FBS) and 1% penicillin (100 UI/ml)-streptomycin (100 mg/ml). After 24 h, cells were treated with 40 ng/ml of soluble recombinant human TNF-α and IFN-γ (Preprotech) and at different time points (6, 24, 48, and 72 h) cells were harvested for flow cytometry phenotyping and RNA analysis.

### Peripheral blood mononuclear cell (PBMC) assay

Human PBMC were isolated from heparinized whole-blood samples, collected at Methodist Hospital Blood Donor Center (IRB-Pro00012800), by using Ficoll density gradient protocol. PBMC proliferation was induced by stimulating cells with 1.5% phytohemagglutinin (PHA; Sigma-Aldrich) in α-MEM standard media. The effect of MSCs grown on the CS on T lymphocyte proliferation was determined in a cell–cell contact setting. To assess the stem cell effects on PBMC proliferation, BMMSC or CPSC were seeded onto CSCL^28^. After 24 h, PBMCs in suspension were stained with BD Horizon Violet Cell Proliferation Dye 450 (VPD450; BD Biosciences) for 25 min, washed in PBS, and added for 72 h to BMMSC or CPSC culture at 10:1 ratio. To evaluate the living cells, staining with Live/DeadViability/Cytotoxicity Kit for mammalian cells (Invitrogen) was performed, following manufacturer’s specifications, and analyzed by flow cytometer^29^.

### Gene expression analysis

Total RNA was isolated using 0.5 ml of Trizol reagent (Life Technologies, ThermoFisher Scientific). For cell cultured in 3D scaffolds, a homogenization step with a PowerGen 125 tissue homogenizer (ThermoFisher Scientific) was performed before adding 1 ml of Trizol. For each sample, RNA concentration and purity were measured using a NanoDrop spectrophotometer (ND1000, NanoDrop, ThermoFisher Scientific). Treatment with DNAse (Sigma-Aldrich) was performed to avoid DNA contamination. cDNA was synthesized from 1 μg of total RNA using a Taqman Reverse Transcription reagent kit (Applied Biosystems, ThermoFisher Scientific). Amplifications were set on plates in a final volume of 10 μl and carried out using TaqMan Fast Advanced MasterMix (Applied Biosystems, ThermoFisher Scientific). The housekeeping markers included in the study were beta actin (*ACTB*; Hs060665_g1) for the 2D differentiation experiments and glyceraldehyde-3-phosphate dehydrogenase (*GAPDH*; Hs02786624_g1), eukaryotic 18S rRNA (*18S*; Hs03003631_g1) and ribosomal protein L13a (*RPL13a*; Hs04194366_g1) for cytokine treatments in 2D and 3D.

### Specific lineage-associated markers

These markers included SRY related high-mobility group box transcription factor (*SOX9*; Hs01001343_g1), type 2 collagen (*COL2A1*; Hs00264051_m1), and aggrecan (*ACAN*; Hs00153936_m1) for chondrogenesis expression. Osteocalcin (*BGLAP*; Hs01587814_g1), Runt-related transcription factor 2 (*RUNX2*; Hs00231692_m1), and alkaline phosphatase (*ALP*; Hs01029144_m1) for osteogenesis expression and leptin (*LEP*; Hs00174877_m1), lipoprotein lipase (*LPL*; Hs00173425_m1) and adiponectin (*ADIPOQ*; Hs00605917_m1) for adipogenesis expression genes.

### Immunosuppression-associated markers

These markers included prostaglandin E synthase (*PTGES*; Hs01115610_m1), prostaglandin-endoperoxide synthase 2 (*PTGS2*; Hs00153133_m1), and indoleamine 2,3-dioxygenase 1 (*IDO1*; Hs00984148_m1)^30,31^.

### 3D collagen-chondroitin sulfate scaffold fabrication

Porous collagen-chondroitin sulfate (CS) scaffolds were fabricated with the freeze-dry technique previously reported^28,32^. Briefly, we prepared an acetic collagen type I slurry (40 mg/ml), which was precipitated to a pH of 5.5 with NaOH (1.67 mM). Chondroitin sulfate (CS; Carbosynth) was added to the collagen solution at a weight molar ratio of 10:1 (CL:CS). After thorough mixing, the final slurry was poured onto a 24-well plate and freeze-dried. CSCL was cross-linked for 4 h at 37 °C using 50 mM 2-(N-morpholino)ethanesulfonic acid, 5 mM 1-ethyl-3-(3-dimethylaminopropyl)carbodiimide (EDC), and 5 mM N-hydroxysuccinimide. CSCL scaffolds were then rinsed twice for 1 h with 0.1 M disodium phosphate and 6 times for 24 h with 2 M sodium chloride; finally, they were rinsed with distilled water to remove residual EDC. Scaffolds were air-dried and sterilized by ultraviolet irradiation for 24 h under a laminar flow hood.

### Confocal microscopy imaging

The cells cultured in 2D or onto 3D scaffolds with or without immunogenic treatment for 72 h were fixed with 4% formaldehyde in PBS. Samples grown in 2D were permeabilized in 0.1% Triton X-100 (Sigma-Aldrich, USA) and blocked in 3% BSA in PBS. Overnight incubation with primary mouse anti-ICAM1 antibody (ab2213; Abcam, UK) diluted 1:250 was followed by goat Anti-Mouse IgG H&L (Alexa Fluor 488) preadsorbed secondary antibody (ab150117; Abcam, UK) diluted 1:500 plus Alexa fluor 647 Phalloidin (A22287; Invitrogen, USA) diluted 1:40 1 h incubation. DAPI (NucBlue, USA) was added to the wells prior to analysis by confocal microscopy. Cells grown onto 3D scaffolds were stained for actin cytoskeleton (1:40 dilution; Alexa Fluor 555 phalloidin, Invitrogen, USA) and nuclear DNA (5 μM DRAQ5; eBioscience, USA). Images were captured on a confocal laser microscope (A1 Confocal Microscope, Nikon Instruments). Attached antibodies fluorescence was quantified in the confocal images using corrected total cell fluorescent intensity plug in the Image J software, and the results were statistically analyzed.

### Statistical analysis

Statistical analysis was performed by using GraphPad Instat 3.00 for Windows (GraphPad Software). Three replicates for each experiment (cell doubling, cell proliferation, cell cytotoxicity, gene expression and PBMC test) were performed, and the results are reported as mean ± SD, with *p* ≤ 0.05 used as a threshold for significance. One-way analysis of variance by the Student–Newman–Keuls multiple comparison test was used.

## Results

### BMMSC and CPSC isolation, CFU, self-renewal capacity and viability analysis

**Figure 1:**
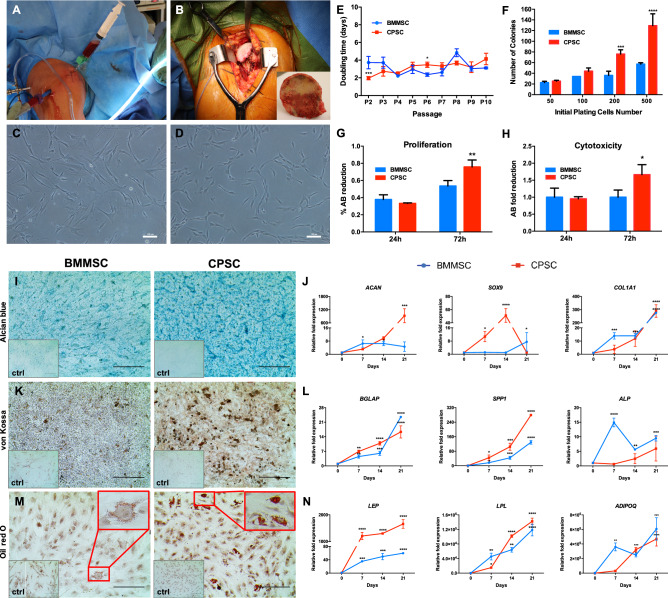
BMMSC and CPSC isolation and stem cell characterization. (**A**) Bone marrow aspiration procedure from a proximal humeral needle insertion site. (**B**) Shoulder joint replacement surgery with the humeral head collected for the biorepository bank. (**C**) BMMSC in a culture plate after their isolation. (**D**) CPSC isolated from joint cartilage and cultured in vitro in fibronectin-coated plates. (**E**) Cell proliferation analysis of in vitro cultured BMMSC and CPSC, expressed as doubling times at different passages (from P2 to P10). (**F**) Colony forming efficiency of BMMSC or CPSC collected at P2 and plated for 14 days at different cell numbers (50, 100, 200 and 500) (**G**) Cell proliferation, evaluated by AlamarBlue assay, of BMMSC and CPSC at different time points. (**H**) Cytotoxicity of CPSC compared to BMMSC in culture for 72 h. (**I**) Representative images of alcian blue glycosaminoglycan staining of chondrogenesis induced BMMSC and CPSC at 21 days. (**J**) Relative gene expression of chondrogenesis markers *ACAN, SOX9* and *COL1A1* at 0, 7, 14 and 21 days in induced cells compared to their control BMMSC (blue) and CPSC (red). (**K**) Representative images of von Kossa mineral deposition staining of osteogenesis induced BMMSC and CPSC at 21 days. (**L**) Relative gene expression of osteogenesis markers *BGLAP, SPP1* and *ALP* at 0, 7, 14 and 21 days in induced cells compared to their control BMMSC (blue) and CPSC (red). (**M**) Representative images of Oil red O lipid deposits stained in adipogenesis induced BMMSC and CPSC at 21 days. (**N**) Relative gene expression of adipogenesis markers *LEP*, *LPL* and *ADIPOQ* at 0, 7, 14 and 21 days in induced cells compared to their control BMMSC (blue) and CPSC (red). (**O**) Flow cytometry percentages of negative anti-hematopoietic lineage cocktail (Neg. cocktail^−^) and triple positive anti CD90, CD105 and CD73 through different passages. Scale bar represents 200 μm. Asterisk represents statistically different means between groups (n = 3), *p < 0.05, **p < 0.01, ***p < 0.001, **** *p* < 0.0001.

BMMSC and CPSC populations were isolated from primary human samples collected at Houston Methodist Orthopedic and Sports Medicine clinics (IRB CR00006624) (Fig. 1A,B). No significant differences were observed in donor age between the 19 male and 12 female donor cohorts (*p* < 0.05; T test). When cultured in vitro, both cell subsets adhered to plastic culture plates and displayed a fibroblastic-like shape resembling MSC morphology (Fig. 1C,D). Cell proliferation analysis indicated that CPSC present a 1.89-fold faster cell doubling time at initial passage compared to BMMSC (P2 culture, *p* < 0.0001, Fig. 1E). This property was lost over time. Indeed, over 10 in vitro culture passages BMMSC and CPSC displayed similar cell doubling times accounting for 3.21 ± 0.8 and 3.16 ± 0.7 days, respectively (Fig. 1E). We then assessed the colony forming efficiency. At low cell number (50 or 100 plated cells), CFU analysis did not reveal any difference between BMMSC and CPSC (Fig. 1F). On the contrary, when we increased the number of cells to 200 and 500, a higher colony forming efficiency was observed in CPSC compared to BMMSC (2.11-fold, *p* < 0.001 and 2.25-fold, *p* < 0.0001 respectively). Accordingly, AlamarBlue reduction assays revealed a significant 1.41 ± 0.12-fold increase (*p* = 0.0041) in CPSC proliferation rate after 72 h in 2D culture (Fig. 1G), despite a significant 1.66 ± 0.24-fold (*p* = 0.045) increase in CPSC cellular cytotoxicity, compared to the BMMSC counterpart (Fig. 1H). In summary, these results indicate that CPSC and BMMSC are endowed with similar morphological and proliferative features.

Then we characterized the immune phenotype of BMMSC and CPSC evaluating the expression of the stem-like cell surface markers CD90, CD105 and CD73 and the concomitant absence of hematopoietic stem cell (HSC) markers CD34, CD45, CD11b, CD19 and HLA-DR. Flow cytometry analysis of BMMSC over the first three in vitro passages showed consistent antigen expression profiles with high percentages of triple positive cells (99.1%, 99.3% and 98.4% at P1, P2 and P3, respectively) and no expression of HSC negative markers (Fig. 1O). Similar phenotype was observed in CPSC, with 99.5%, 98.7% and 98.8% triple positive cells at the reference passage points. Interestingly, we did not detect any significant alterations in BMMSC or CPSC morphology over the in vitro culture passages (P0-P3; Supporting Information Fig. 1).

### Multilineage differentiation and molecular characterization

We assessed the stem-like features and trilineage differentiation ability of BMMSC and CPSC. First chondrogenic differentiation was induced as detailed in Materials and Methods. Alcian Blue staining revealed that both cell populations showed pronounced glycosaminoglycan deposition after 21 days, in comparison to untreated controls (Fig. 1I). In line, the mRNA expression levels of chondrogenic differentiation-associated genes increased over time in both BMMSC and CPSC (Fig. 1J; *p* < 0.05). In BMMSC, COL1A1 levels from 13.95 ± 1.4 at day7 reached 276.54 ± 2.2 at day 21; similarly, SOX9 increased from 1.18 ± 0.2 to 5.27 ± 3.2 in two weeks. In CPSC, COL1A1 from 3.7 ± 1.8 at day 7 reached 289.9 ± 27.4 at day 21. SOX9 instead increased from 10.9 ± 1.7 at day 7 to 51.1 ± 7.3 at day 14 and then decreased to 1.06 ± 0.38 at day 21. Significantly increased ACAN gene expression was observed in the BMMSC treated samples after 7-days treatment in BMMCS, however, in the CPSC samples, significantly increased ACAN expression was only observed at day 21 (745.4 ± 69.3-fold, *p* < 0.001).

**Figure 2:**
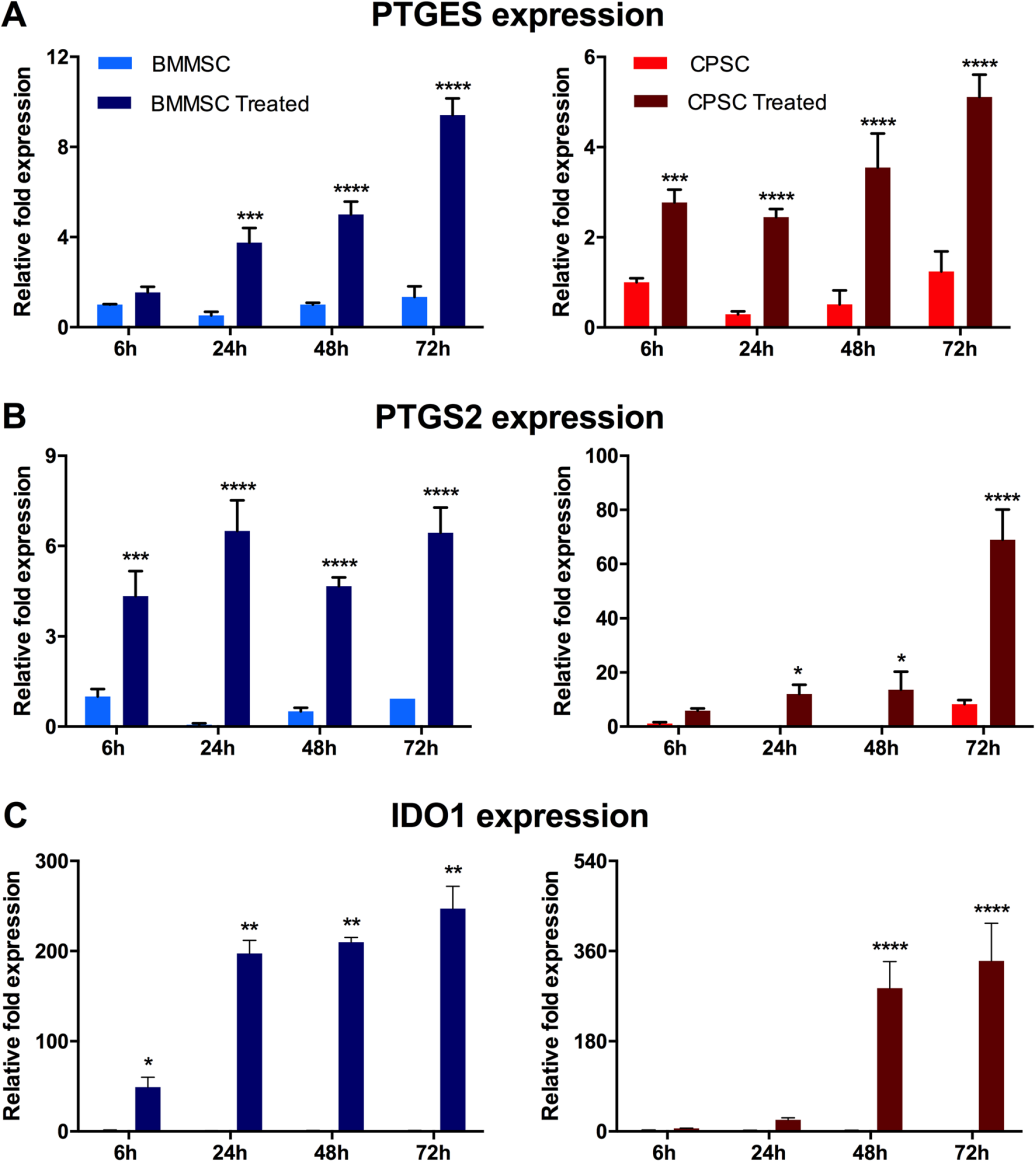
BMMSC and CPSC secrete cytokines after immunogenic treatment. (**A**–**C**) Relative expression of PTGES, PTGST2 and IDO1 at after 6 h, 24 h, 48 h and 72 h of treatment with 40 ng/ml TNFa and 40 ng/ml INFy . Asterisk represents statistically different means between BMMSC (blue) and CPSC (red), (n = 3), *p < 0.05, **p < 0.01, ***p < 0.001, ****p < 0.0001.

Successively, BMMSC and CPSC were cultured with osteogenic media for 21 days. As revealed by von Kossa staining, both cell subsets exhibited mineral depositions over time, with a similar intensity increase (Fig. 1K and Supporting Information S2). This result was supported by the mRNA analysis that demonstrated an overall increase of osteogenic-associated gene expression in both BMMSC and CPSC (Figs. 1L, 2). However, BMMSC showed significantly higher expression of ALP at all time points evaluated, compared to CPSC (14-fold, fivefold, and twofold higher at 7, 14 and 21 days respectively). On the contrary, CPSC expressed higher levels of SPP1 compared to BMMSC (33.09 ± 12.8 vs. 16.9 ± 0.25 at day 7; 104.8 ± 11.1 vs. 43.9 ± 4.7 at day 14 and 279.1 ± 3.18 vs. 129.3 ± 6.12 at day 21). BGLAP expression, instead, increased in both cell types from 4.48 ± 0.4 at day 7 to 23.52 ± 0.19 at day 21 in BMMSC and from 6.95 ± 0.52 to 16.36 ± 2.06 in CPSC (Fig. 1L; *p* < 0.05).

Similarly, in the presence of adipogenic media, both cell populations formed fat deposits, as shown by Oil Red O staining. Although it was not quantified, CPSC displayed seemingly increased fat deposit vacuoles compared to BMMSC (Fig. 1M). This result was supported by the evaluation of *LEP*, *LPL* and *ADIPOQ* expression over the 21 days culture period (Fig. 1N; *p* < 0.05). In detail, at all the analyzed time points CPSC demonstrated higher levels of *LEP,* compared to BMMSC (1203.5 ± 79.3 vs. 35.3 ± 0.9 at day7; 1300.2 ± 12.9 vs. 49.7 ± 5.5 at day 14 and 1657.9 ± 93.4 vs. 59.73 ± 0.26 at day 21; Fig. 1N, *p* < 0.05).

### Immunosuppressive potential

In order to assess the immune tuning capability of BMMSC and CPSC, mRNA expression levels of *PTGES*, *PTGS2* and *IDO1* was evaluated in the two cell subtypes after 24 h of in vitro standard culture conditions followed by 72 h of stimulation with TNFα and INFγ. PTGES and IDO represent the key players in the suppression of inflammatory cascade during chronic inflammation^33,34^. We demonstrated PTGES, controlled by PTGS2, triggers a PGE2-mediated TNFα down-regulation and IL10 up-regulation^30,31^. Moreover, PTGS2 is also responsible for the expression of several other specialized pro-resolving inflammatory mediators (such as PGI2) and it is commonly monitored in inflammatory research studies. Similarly, IDO, through INFγ response elements, inhibits innate and adaptive immunity and induces tolerance. new insights in chronic inflammation and cancer have been described^35–37^.

Previous dose–response titration identified optimal TNFα (40 ng/ml) plus INFγ (40 ng/ml) combination treatments, associated with no significant cytotoxicity at 24 and 72 h (Supporting Information Fig. S3). After 24 h of treatment, treated BMMSC exhibited significant increased PTGES expression compared to the untreated control (3.75 ± 0.20-fold, *p* < 0.001). This difference further increased over time (5.01 ± 0.46-fold and 9.41 ± 0.60-fold at 48 and 72 h respectively, *p* < 0.0001; Fig. 2A, left panel). CPSC, instead, showed a significantly increase of PGTES expression after only 6 h of treatment (2.77 ± 0.23-fold compared to the control; *p* < 0.001) and maintained the increased expression at 24 h, 48 and 72 h (2.45 ± 0.14-fold, 3.54 ± 0.62-fold and 5.11 ± 0.40-fold respectively, *p* < 0.0001; Fig. 2A, right panel).

Similarly, compared to the untreated control, *PTGS2* relative expression consistently increased at all time points in BMMSC. In contrast, CPSC demonstrated a significant increase in *PTGS2* only after 24 h of treatment 48 h (12.02 ± 2.76-fold, 13.61 ± 5.46-fold at 24 and 48 h respectively, *p* < 0.05; 68.96 ± 9.13-fold at 72 h, *p* < 0.0001) (Fig. 2B). *IDO1* expression exhibited a different profile in BMMSC and CPSC (Fig. 2C). Alternatively, in BMMSC *IDO1* significantly increased after only 6 h of treatment (49.18 ± 8.92, *p* < 0.01), and further increased at 24, 48 and 72 h (197.3 ± 10.41-fold, 210.01 ± 3.71-fold and 247.10 ± 20.17-fold respectively, *p* < 0.0001). On the contrary, CPSC showed no significant change in *IDO1* expression compared to the control, at 6 and 24 h (*p* > 0.05). Only after 48 and 72 h of treatment did *IDO1* expression increase significantly compared to the control (286.10 ± 43.16-fold and 339.31 ± 61.56-fold respectively, *p* < 0.0001).

In treated BMMSC and CPSC we also evaluated the protein expression of the stem cell surface markers CD34, CD45, CD11b, CD19, HLA-DR, CD90, CD105 and CD73. Flow cytometry analysis revealed that treatment with TNFα and INFγ not only altered the morphology of both cell subsets but was also associated with a time-dependent increase of CD73 mean fluorescence intensity, without affecting the percentage of the stem-like cell subset (CD34^neg^/CD45^neg^/CD11b^neg^/CD19^neg^/HLA-DR^neg^/CD90^pos^/CD105^pos^/CD73^pos^) (Supporting Information Fig. S4).

**Figure 3:**
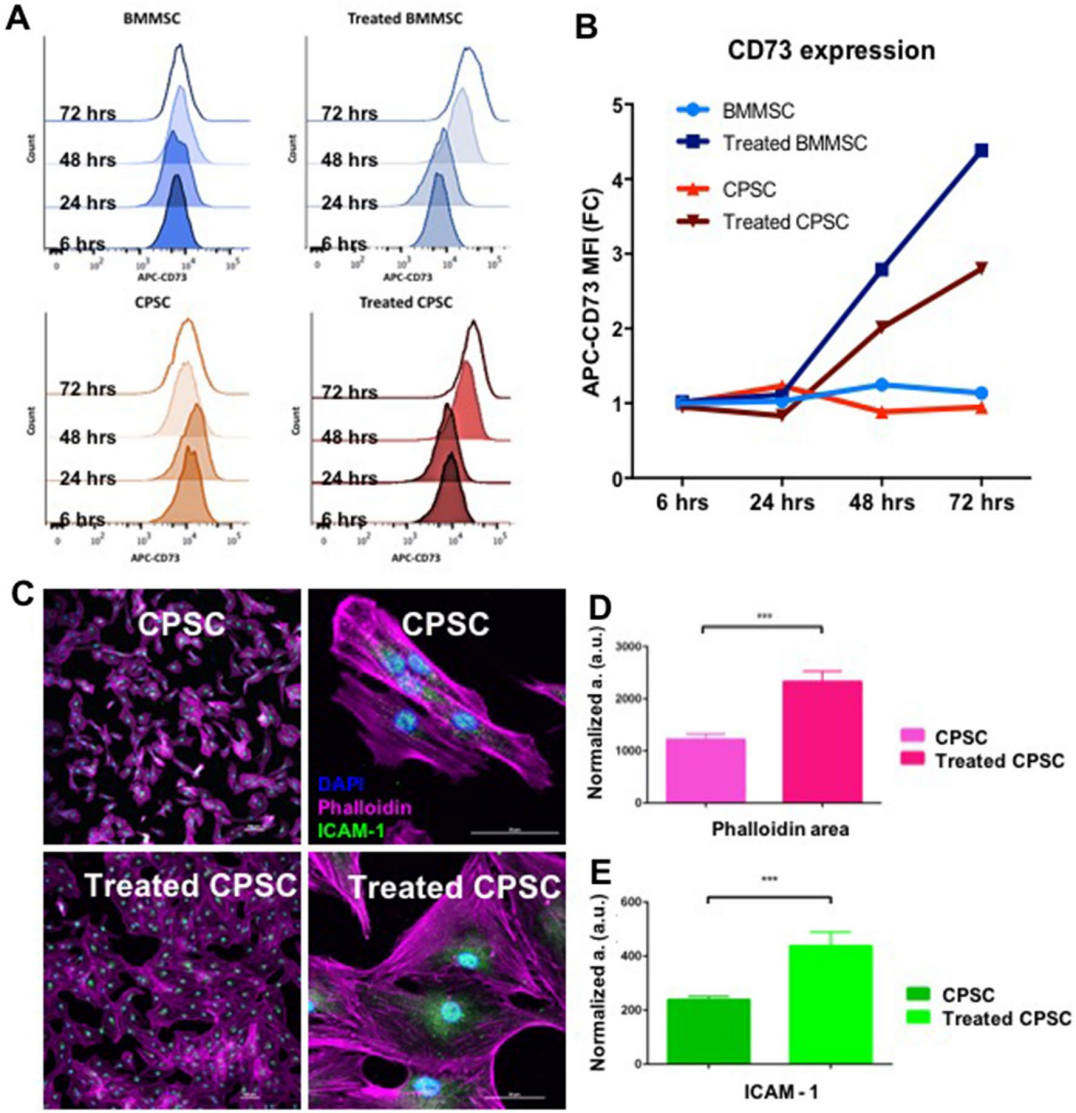
(**A**) Number of events against APC-CD73 fluorescence intensity histogram displaying the shift of stained cells after 6 h, 24 h, 48 h and 72 h of treatment compared to untreated controls. (**B**) BMMSC and CPSC relative APC-CD73 mean fluorescence intensities (MFI) compared to untreated control. (**C**) CPSC representative immunofluorescent confocal microscopy images of actin filaments labelled with Phalloidin (pink), anti-ICAM antibody (green) and cell nuclei with DAPI (blue) after treatment compared to untreated controls. Scale bars represent 100 μm (left) and 50 μm (right). (**D**) Inflamed CPSC phalloidin fluorescence area quantification compared to untreated control cells. (**E**) Inflamed CPSC anti-ICAM1 fluorescence area quantification compared to untreated control cells. Results were tested for significance using a student t-test between groups from a minimum of 6 images with three biological replicates. *** was used for *p* values lower than 0.001.

Consistent with the immune suppressive marker expression, cellular immune potential was evaluated in a lymphocyte reaction assay. Flow cytometry analysis demonstrated the ability of both BMMSC and CPSC to inhibit the proliferation of PHA-stimulated PBMC following 3 days of co-culture (Fig. 3B,C). The presence of the two cell subsets was associated with a reduction in the relative median fluorescence intensity (MFI) from 1 to 0.52 ± 0.24 and 0.79 ± 0.7 (BMMSC and CPSC respectively; *p* < 0.05) (Fig. 3A). PHA-treated PBMC displayed a significant decrease in Calcein AM expression, suggesting decreased cell viability (Fig. 3B; *p* < 0.0001). However, no difference was observed when PHA-activated PBMC were co-culture with BMMSC and CPSC or left alone (Fig. 3B, *p* > 0.05).

### 3D biomimetic scaffolds enhance BMMSC and CPSC immunomodulation

**Figure 4:**
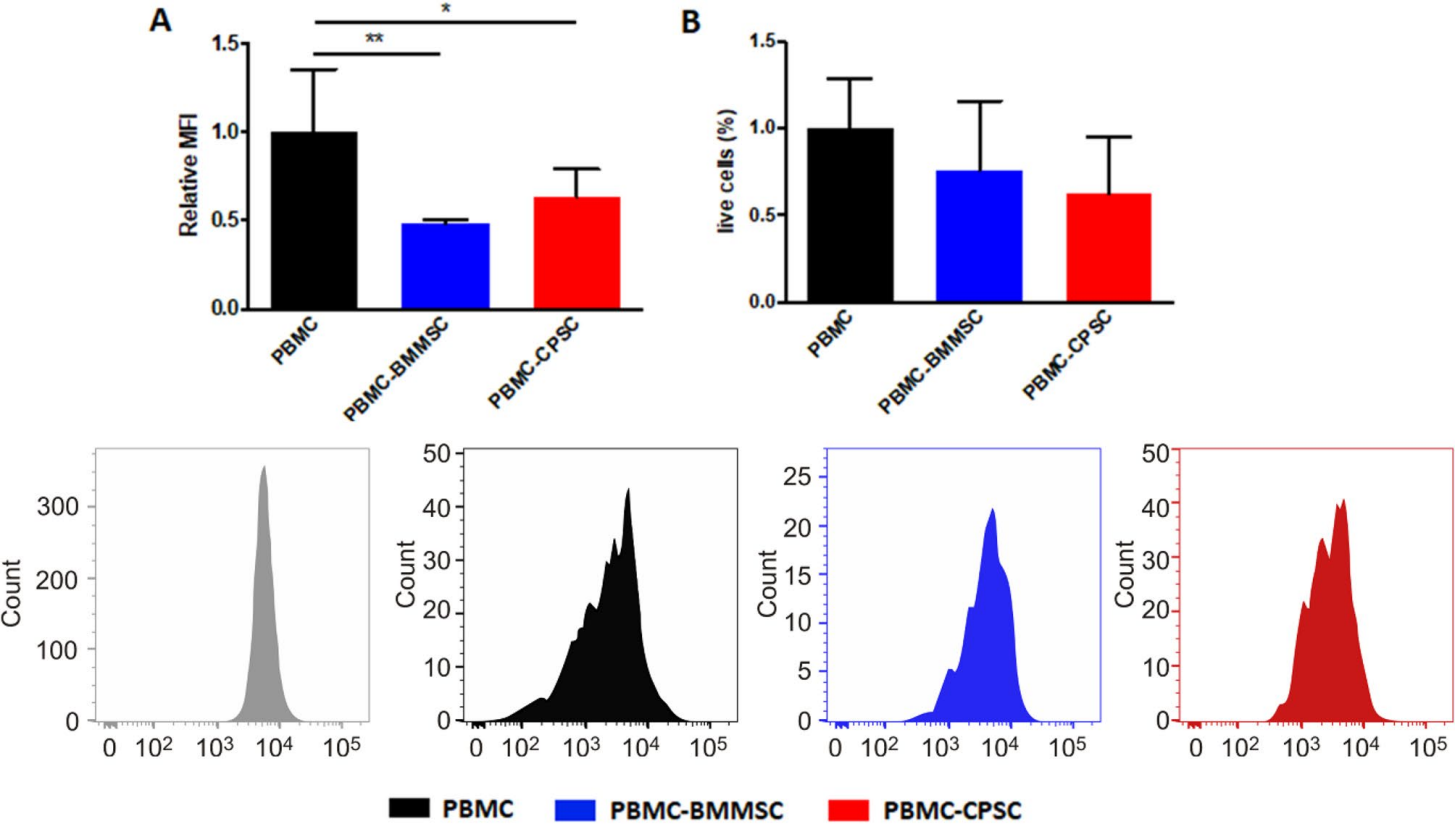
BMMSC and CPSC inhibit proliferation of PBMC. (**A**) CellTrace violet cell proliferation stained PBMC after 72 h co-culture with BMMSC or CPSC. (**B**) Cell viability of calcein stained PBMC after activation and co-culture with BMMSC or CPSC for 72 h. (**C**). Number of events against CellTrace violet fluorescence intensity histogram of activated PBMCs alone or co-cultured with either BMMSC or CPSC (T0 control in grey). Asterisk represents statistically different means between groups (n = 3), *p < 0.05, **p < 0.01, ***p < 0.001, ****p < 0.0001.

We have previously demonstrated an increased immunosuppressive capacity of stem cells when cultured in a 3D setting using a biomimetic collagen-made scaffold^28,38^. Thus, we hypothesized that a porous scaffold would enhance the immunomodulatory properties of human BMMSC and CPSC. To test this hypothesis, BMMSC and CPSC were cultured within collagen-chondroitin sulfate scaffolds. In this culture condition, the two cell subsets demonstrated a clear ability to invade and proliferate within the scaffold structure, both in control media and in the presence of inflammatory cytokines (Fig. 4A and Supporting Information Fig. S5, Videos 1, 2, 3).

To assess the immune tuning capacity of the cells in the 3D culture condition, BMMSC and CPSC seeded in 3D scaffolds were treated with TNFα (40 ng/ml) plus INFγ (40 ng/ml). RNA expression levels of *PTGES*, *PTGS2* and *IDO1* were analyzed at 6, 24, 48 and 72 h. As represented in Fig. 4B, BMMSC showed significantly increased *PTGES* expression at 48 h and 72 h (66.16 ± 6.01-fold and 71.11 ± 9.10-fold, respectively, *p* < 0.0001) compared to untreated or treated cells in 2D culture setting. CPSC displayed 25.16 ± 3.41-fold increased expression at 24 h (*p* < 0.0001), a 18.65 ± 2.57-fold increase at 48 h, and a 63.22 ± 6.63-fold increase at 72 h (Fig. 4B; *p* < 0.0001).

Compared to 2D culture, *PTGS2* relative expression in BMMSC increased significantly at 48 and 72 h (*p* < 0.0001). On the contrary, CPSC treated in 3D culture, showed an increase in relative expression of *PTGS2* only at 72 h (731.00 ± 224.52-fold respectively, *p* < 0.0001). Similarly, *IDO1* expression increased in BMSC at 24, 48 and 72 h, whereas increased in CPSC only after 48 and 72 h (*p* < 0.0001).

Compared to the 2D culture, the 3D scaffold did not induce any changes in BMMSC and CPSC morphology or stem cell marker expression following cytokine treatment. This supports the hypothesis that the stem/progenitor cell phenotype is stable in 3D culture (Supporting Information Video 4).

**Figure 5:**
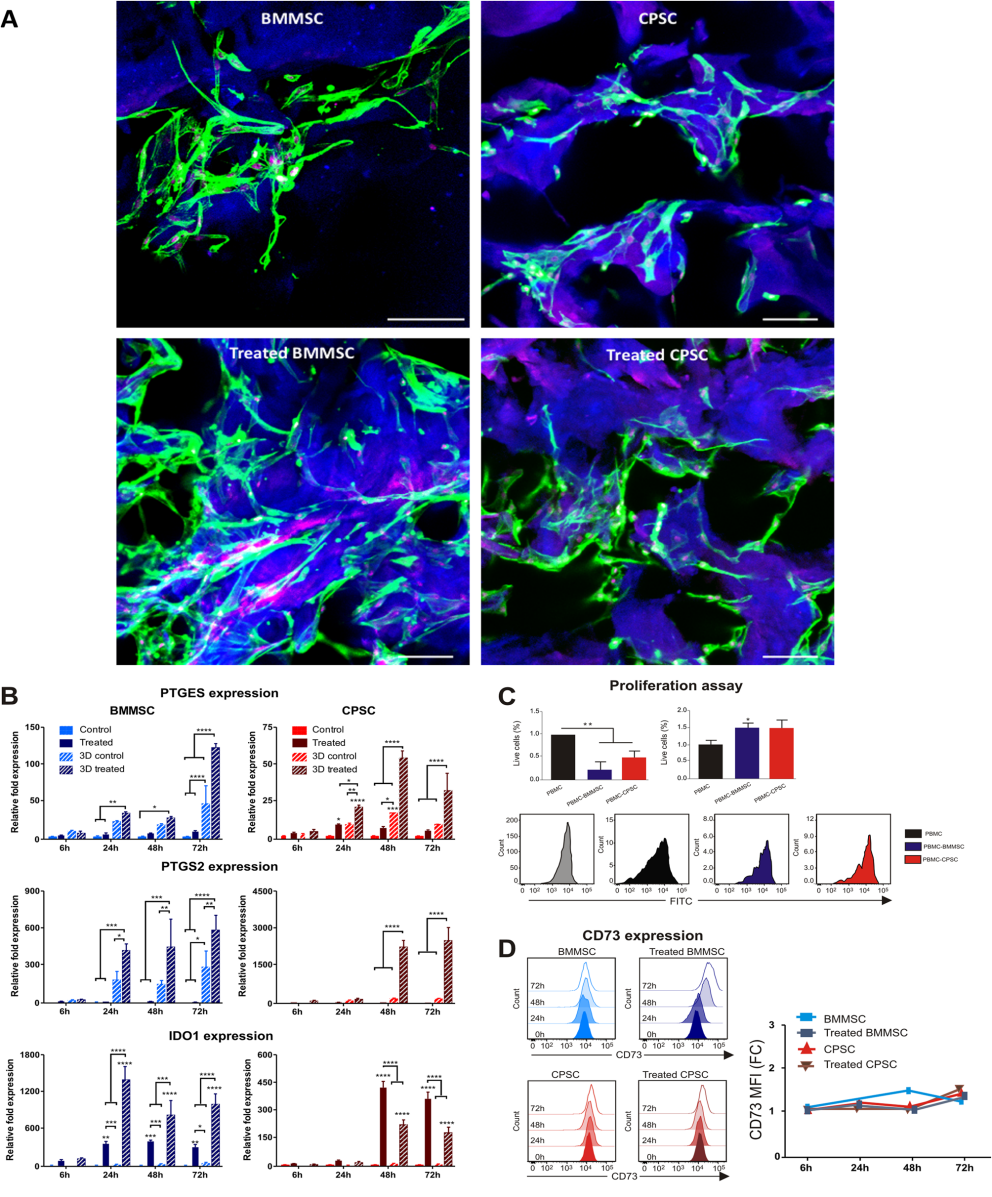
3D biomimetic scaffold enhances immunomodulation of BMMSC and CPSC. (**A**) Confocal microscopy images of viable stem cells colonizing the biomimetic scaffolds at 72 h using Phalloidin (Alexa fluor 555) and Draq5 (far-red) staining, and scaffold autofluorescence (blue). (**B**) Relative expression of PTGES, PTGST2 and IDO1 at 6 h, 24 h, 48 h and 72 h after treatment with 40 ng/ml TNFa and 40 ng/ml INFy. (**C**) PBMCs proliferation assay after activation and co-culture with BMMSC or CPSC. (**D**) CD73 expression profiles shift. Asterisk represents statistically different means between BMMSC (blue) and CPSC (red), (n = 3), *p < 0.05, **p < 0.01, ***p < 0.001, ****p < 0.0001.

To support the immune potential of 3D-cultured BMMSC and CPSC, we then performed a lymphocyte reaction assay. Both cell subsets were able to inhibit the proliferation of PHA-stimulated PBMC following 4 days co-culture, as demonstrated by 0.2 ± 0.18 and 0.52 ± 0.12-fold reduction of MFI BMMSC and CPSC, respectively (Fig. 5C; *p* < 0.01). Flow cytometry analysis of the cells isolated from the 3D experiment revealed that cell morphology, as well as CD73 expression, had not been affected by the inflammatory condition (Fig. 5D).

## Discussion

This study demonstrates that CPSC’s retain an anti-inflammatory, immune modulatory capacity in vitro*.* When compared directly to gold standard BMMSC cell populations, CPSC cells demonstrated similar morphological, proliferative and multilineage differentiation capacity in 2D culture. Similarly, both cell types demonstrated immune suppression marker expression profiles and a clear ability to modify immune cell potential using a lymphocyte reaction assay. Using an advanced 3D collagen-CS scaffold model mimicking the chondrogenic niche, CPSCs and BMMSCs showed enhanced immunomodulatory capacity. Successful utilisation of CS-collagen materials for advanced 3D culture enhances our understanding of immune tuning capacity of resident CPSCs in vitro, providing supportive evidence for their potential to augment initial inflammatory responses shown to be critical to the development of OA^25^.

CPSC and BMMSC isolated from the cartilage and bone marrow, respectively, of orthopedic patients undergoing total-shoulder replacement were thoroughly characterized for their morphological and proliferative characteristics in vitro. Results indicated that even though CPSC presented the same phenotype and similar spindle-like shape of BMMSC^39^, they appeared more rounded, probably due to a pre-commitment phenotype (see Fig. 1C in comparison with 1D). In addition, while the expression of *SOX9* and *COL1A1* was similar to BMMSC, CPSC displayed a higher level of proteoglycan deposition combined with an increased *ACAN* RNA expression, suggesting an advanced state of cellular differentiation toward chondrogenesis. Differences in the cell line’s phenotype might be due to the osteoarthritic nature of the AC samples used in this study. Interestingly, increased metabolism and abnormal matrix remodeling gene expression has been shown in previous studies in cartilage tissue from OA patients^40^. In addition, the osteogenic properties of CPSC were similar to BMMSC, but CPSC revealed higher intensity areas of Von Kossa staining and expression levels of *SPP1*. This result is supported by other studies that have demonstrated that in OA chondrocytes, a high level of mRNA expression of *SPP1* is associated with the pathogenesis of OA^41^. However, other CPSC studies did not report a similar increase^21^. This may suggest that CPSC samples may maintain molecular phenotypes consistent with chondrocytes isolated from OA patients, a point that could be clarified by the inclusion of healthy human sample controls. These samples however are difficult to obtain.

In addition, CPSC seemingly demonstrated increased volume of the fat deposit vacuoles (as observed in Oil Red O staining), as well as overexpression of *LE*P during CPSC adipogenic differentiation, previously reported as a signal of OA^42^. From the differentiation analysis, it seems that the CPSC may maintain an “inflammatory commitment”, similar to that observed in MSCs^43^, even if they expressed the same stem cell markers as BMMSC. In fact, in aged-related models, as OA, BMMSC preferentially shift their differentiation potential from osteogenic to adipogenic lineage. These results suggest a similar behavior for CPSC.

This phenotype has been described as the ability to sustain metabolism and secretion upon inflammation exposure. Thus, we tested CPSC immune regulatory function following inflammation with TNFα and INFγ. Inflamed CPSC changed phenotype presenting a higher normalized cell area, and an increase ICAM-1 protein expression, as previously shown to be closely related to the immune activity of MSC. A phenotype change in MSC has already been reported by Klinker et al.^44^, demonstrating that inflammatory treatment with INFγ was able to induce a morphological change in MSCs, related to their immunosuppressive potential, thus supporting our observations. Similarly, other studies have reported the overexpression of ICAM and its link to an increased MSC immunomodulation activity is due to its involvement in the process of homing^45^. The process of homing for MSCs refers to their potential to migrate at the site of inflammation, similar to leucocytes and exploit their immunosuppression potential at the arrival site^46^. Overexpression of ICAM found in MSCs improved their therapeutic effect in vivo^47^. Therefore, we conclude that the overexpression of adhesion proteins is most likely due to CPSCs being involved in a ‘homing’ process inside the cartilage tissue, a process specific to immunomodulatory cells^48^.

Moreover, in the inflamed in vitro setting, CPSC showed similar immunosuppressive potential to BMMSC. Downregulation of *IDO* at acute time points (6 h and 24 h, Fig. 2), as observed in BMMSCs, may be suggestive of a slower activation of the pathway in CPSCs. This impairment was not related to any cytotoxicity (S2) or any differentiation/activation induced by the treatment (S3). In fact, CPSC maintained their stem cell markers throughout the duration of treatment. Interestingly, flow cytometry analysis of the 2D cultures revealed an increase in CD73 mean fluorescence intensity, in both inflamed BMMSCs and CPSCs. Tan et al. observed that the presence of a subpopulation of MSCs with increased CD73 expression was associated with better cardiac repair, as a result of involvement in the regulation of local immune response through purinergic signaling^49^. In addition, they demonstrated that this high CD73-expressing MSC population was able to recruit CD11b+/CCR2+ macrophages with an anti-inflammatory profile. In line with this study, Shin et al. included CD73-enriched MSC within a hydrogel, thus increasing the bioavailability of extracellular adenosine and reducing the cardiac immune response^50^. In addition, Antonioli et al. reported that an increased CD73 expression caused an ‘immunological switch’ toward an anti-inflammatory states^51^.

CPSC were also evaluated for their immunosuppressive capacity in 2D co-culture with immune cells (PBMC). Similar to BMMSC, CPSC were able to impair the proliferation of the mononucleated cells after 72 h (Fig. 4). This data represents the first positive indication of the immunomodulatory potential of articular chondroprogenitors. The data is similar to that of BMMSCs, indicating that CPSCs may be a suitable source for future cell therapy in cartilage regenerative approaches.

Finally, we exploited a 3D biomimetic in vitro model to assess the immune tuning capacity of the construct to define acellular repair strategies to augment current state of the art approaches (Fig. 5). As reported elsewhere, a 3D structure, which mimics the natural cell environment, can affect the immunosuppressive potential of a specific cell type^52^. In order to evaluate the therapeutic potential of a cellular source in a 3D environment, BMMSCs and CPSCs were cultured in a collagen-based scaffold, characterized elsewhere^28,32^. Results indicated that both the cell subsets maintained their stem cell markers and immunomodulatory potential in this setting. Compared to the 2D culture, however, the 3D environment highly improved the expression of the immunomodulatory genes in both BMMSC and CPSC. On the other hand, 3D culture was not associated with changes in cell morphology or CD73 expression, even with immunogenic treatment. Several factors could be responsible for this difference in CD73 overexpression^44,53^. We hypothesize that in the 3D environment, the presence of extracellular AMP, an important substrate for enzymes, could be less accessible than in the 2D setting. Another hypothesis would be the interaction of chondroitin sulfate with CD73 in the purinergic pathway. Interestingly, Cappellari et al. described the modulation of CD73 enzymatic activity by CS in glioma cells^54^. Similarly, Viera et al. showed 5′ nucleotidase inhibition by CS and HS in liver extract preparations in a dose dependent manner^55^. In conclusion, even though further experiments are needed to investigate this aspect, our results suggest that 3D biomimetic scaffolds and their combination with chondroprogenitor stem cells could become the optimal route for further exploration. The combination of a 3D scaffold and CPSC could maximize the immunomodulatory potential of each individual component, and synergistically improve tissue-engineering approaches. Moreover, the varying behavior of cells in the 2D versus the 3D environment confirms the crucial importance of evaluating the immunosuppressive potential in a 3D setting for applications prior to applying them in the clinical setting.

## Conclusion

By releasing biomolecules that directly targets cells of the innate and adaptive immune system, MSC show an incredible anti-inflammatory effect making them a perfect candidate for regenerative therapies. CPSCs are a potential alternative to BMMSCs currently exploited in several therapeutic approaches. Here, for the first time we demonstrated the immunosuppressive potential of articular chondroprogenitors isolated from OA patients. CPSC seemingly demonstrate higher proliferation rate and higher propensity toward chondrogenesis compared to BMMSC. Our results outline a new immunomodulatory trend for acellular and cellular approaches that can complement current state of the art clinical approaches for OA repair^55^.

Previously proven chondrogenic scaffolds, when employed as an advanced 3D cell culture system, further enhance the immunosuppressive potential of both BMMSC and CPSC. Therefore, expanded BMMSCs and CPSC populations may benefit from exposure to the 3D scaffold environment in vitro, prior to implantation and use in the clinical setting. However, current FDA regulations on orthopedic implantable devices do not address this important aspect, which may affect the results of tissue engineering trials^44^. As a result, the study proposes the need for advanced in vitro cell culture analysis methods, to completely evaluate a scaffold’s effect on cell populations within a niche prior to clinical evaluation and implementation.

## Supplementary information

Supplementary Movie 1.

Supplementary Movie 2.

Supplementary Movie 3.

Supplementary Movie 4.

Supplementary Figures.
