# Supplementary material for: Improving the immunosuppressive potential of articular chondroprogenitors in a three-dimensional culture setting
Source: Sci Rep. 2020 Oct 6;10:16610. doi: 10.1038/s41598-020-73188-9 (PMC7538570; doi:10.1038/s41598-020-73188-9)
Supplement: Supplementary file 5 — Supplementary Figures. [file 41598_2020_73188_MOESM5_ESM.pdf]

Title: Improving the immunosuppressive potential of articular chondroprogenitors with a tridimensional culture setting.

Guillermo Bauza[1,2,3], Anna Pasto[2,3], Patrick Mcculloch[3], David Lintner[3], Ava Brozovich[2,3,4], Federica Banche Niclot[2,3,5], Ilyas Khan[1], Lewis W Francis[1], Ennio Tasciotti[1,2,3] and Francesca Taraballi[2,3].*

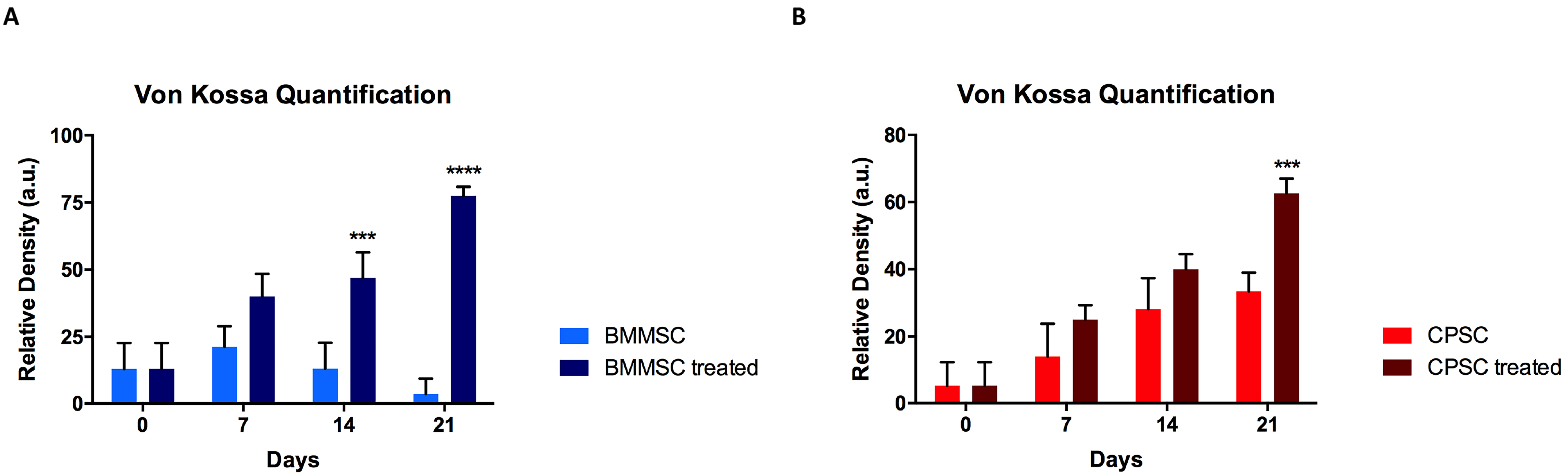

Supporting information figure 1. Von Kossa stained sample images quantification of BMMSC (A) and CPSC (B) populations treated with osteogenic media for 21 days in comparison to untreated controls (n=3), *** p<0.001 and **** p<0.0001.

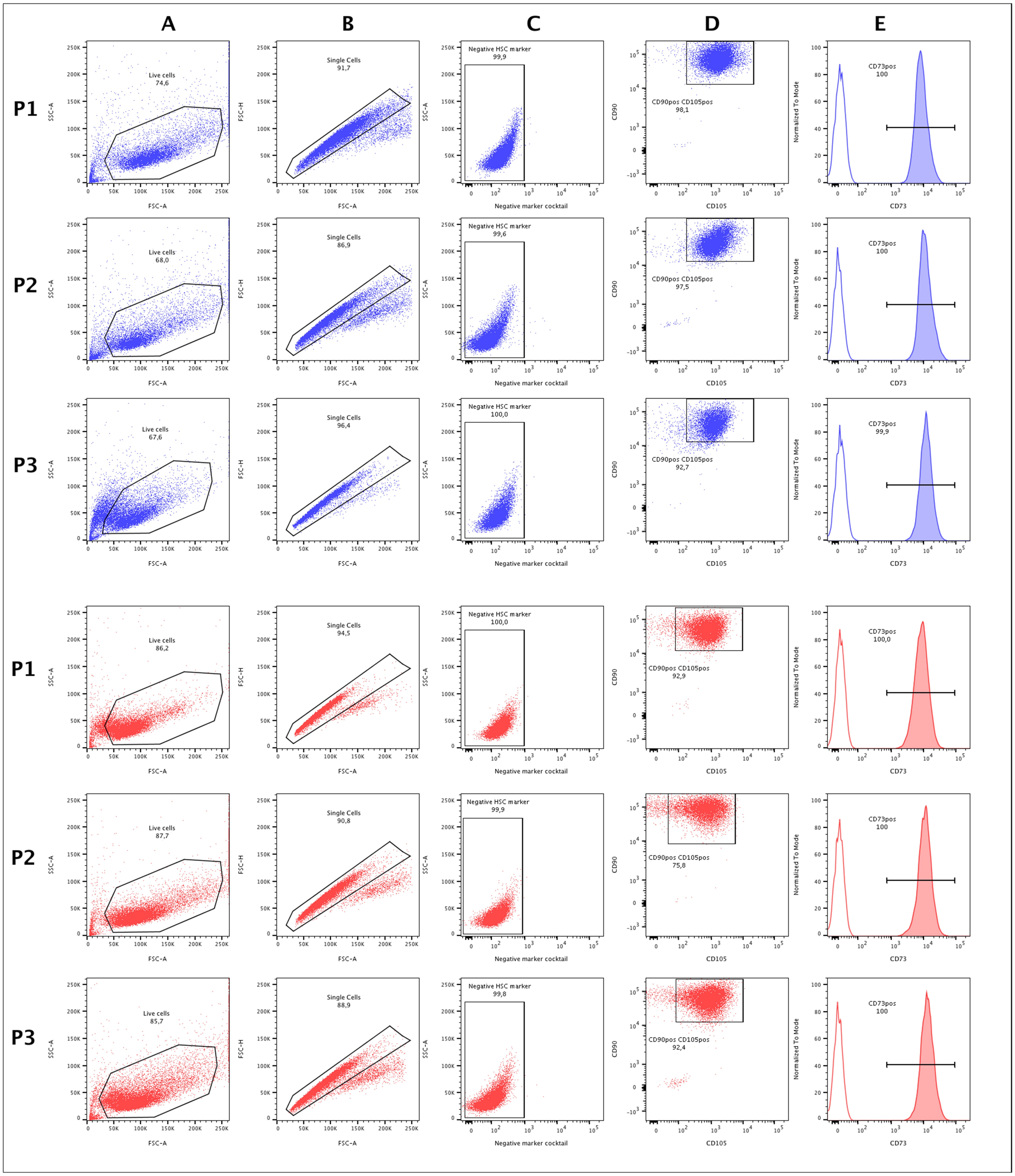

Supporting information figure 2. Immunophenotyping through different passages does not differ significantly between BMSC and CPSC. Gating strategy: Cell morphology defined by side versus forward scatter area **(A)**, singlets identification **(B)**, exclusion of CD34-expressing cells **(C)**, identification of double-positive CD90 and CD105 cells **(D)** and analysis of CD73 expression in the double positive gated cells **(E)** BMMSC (blue) and CPSC (red).

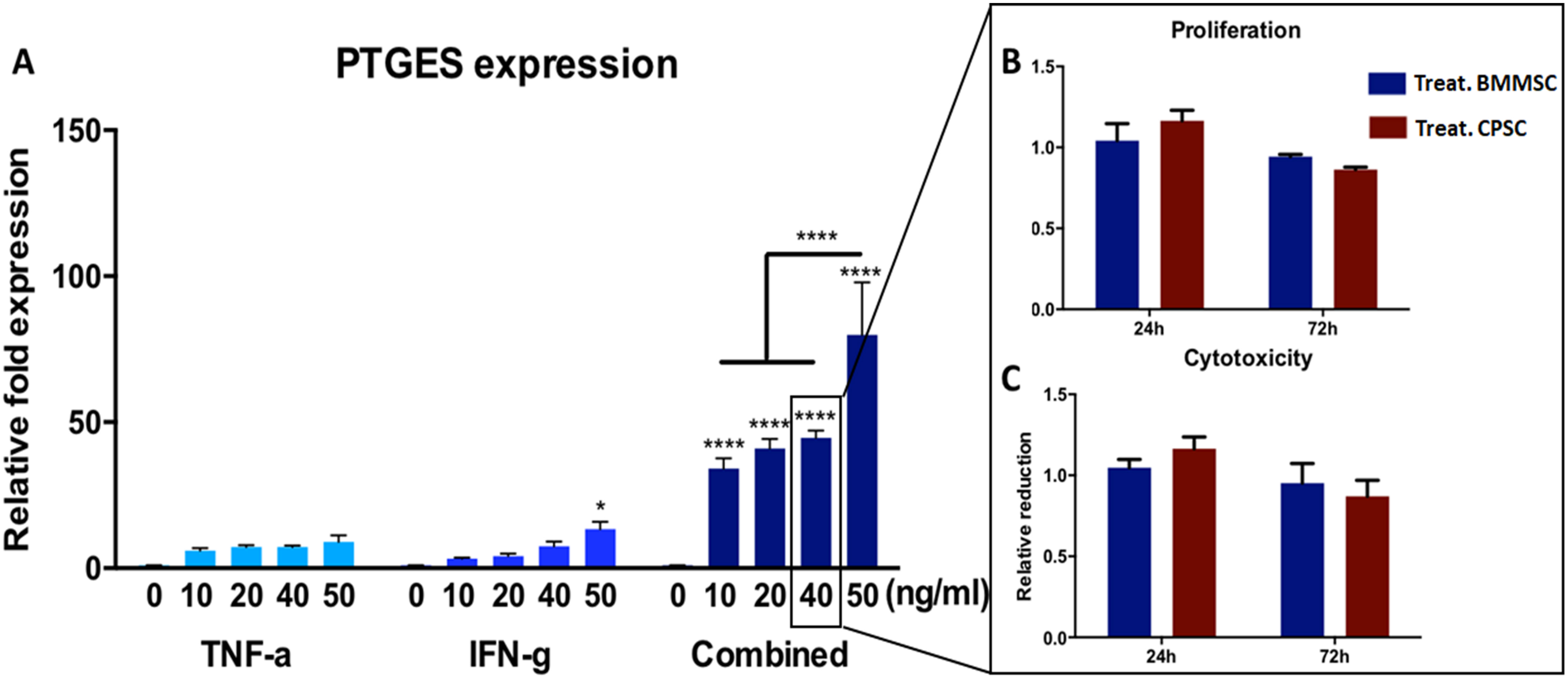

Supporting information figure 3. Treatment optimization. (A) PTGES relative expression of BMMSC treated with increasing quantities of TNFα and INFγ alone or in combination; asterisk represents statistically different means between treated BMMSC and untreated control (n=3), *p<0.05, **p<0.01, *** p<0.001, **** p<0.0001. (B, C) Proliferation (B) and cytotoxicity (C) analysis of BMMSC (dark blue) and CPSC (dark red) at 24h and 72h of treatment with TNFα (40 ng/ml) and INFγ (40 ng/ml). (n=3).

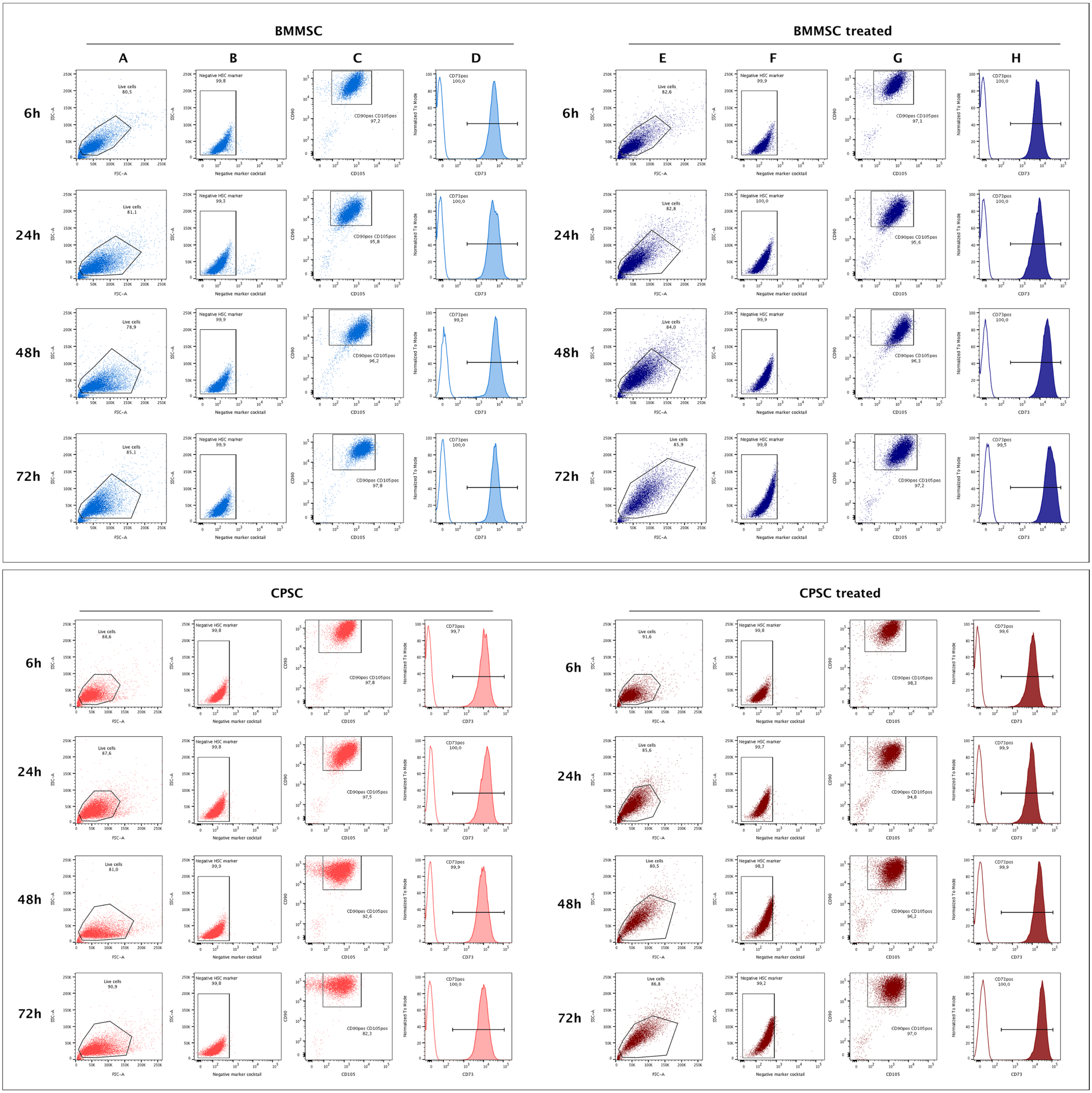

Supporting information figure 4. Stem cells maintain their phenotype during treatment. Gating strategy: Cell morphology defined by side versus forward scatter area (A, E) exclusion of CD45-expressing cells **(B, F),** identification of double-positive CD90 and CD105 cells **(C, G)** and analysis of CD73 expression in the double positive gated cells. BMMSC (blue), treated BMMSC (dark blue), CPSC (red) and treated CPSC (dark red) (n=3), at 6h, 24h, 48h and 72h in culture with normal media or media supplemented with TNFα (40 ng/ml) and INFγ (40 ng/ml).

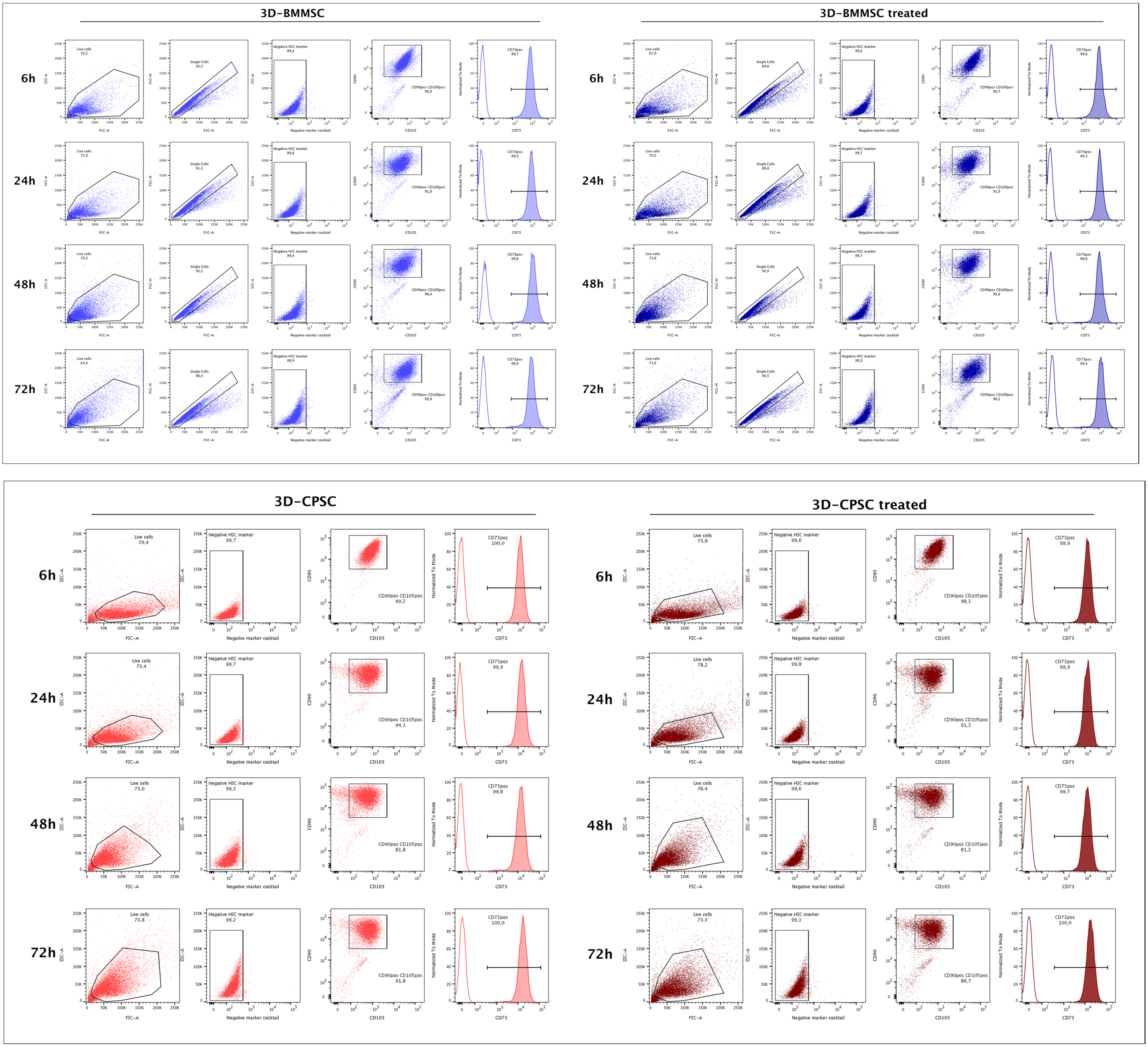

Supporting information figure 5. Stem cells maintain their phenotype during treatment in 3D setting. Gating strategy: Cell morphology defined by side versus forward scatter area (A, E) exclusion of CD45-expressing cells **(B, F),** identification of double-positive CD90 and CD105 cells **(C, G)** and analysis of CD73 expression in the double positive gated cells. BMMSC (blue), treated BMMSC (dark blue), CPSC (red) and treated CPSC (dark red) (n=3), at 6h, 24h, 48h and 72h in culture with normal media or media supplemented with TNFa (40 ng/ml) and INFγ (40 ng/ml).
